# Supplementary material for: Machine learning mathematical models for incidence estimation during pandemics
Source: PLoS Comput Biol. 2024 Dec 23;20(12):e1012687. doi: 10.1371/journal.pcbi.1012687 (PMC11706490; doi:10.1371/journal.pcbi.1012687)
Supplement: Attachment — Submitted filename: Incidence_estimation_REBUTTAL.pdf [file pcbi.1012687.s001.pdf]

Reply to Reviewer #1

COMMENT: **This paper attacks a key problem in tracking infectious diseases—how can we correct for the biases in reported cases (which are often incomplete representations of infections) to better estimate the actual infections circulating in the population. While I do think this study has some potential, there is not sufficient evidence currently to support its main claims. Specifically, I am not convinced that the proposed machine learning method has been shown to improve what is already known from the literature and I think that results have not been demonstrated to be as general as is sometimes claimed. Some substantial additional work is required.**

We appreciate your reading of our manuscript, and are happy that you agree with us on the importance of developing new methodologies for correcting biases in reported cases while tracking infectious diseases. In what follows, we carefully address all your comments, and clarify some misunderstandings that seem to have arisen in your initial assessment of our work. We have also engaged more thoroughly with the related literature.

Moreover, we have now added a new simulation study where we confirm the ability of the BMS to identify the ground-truth models for the rate of reporting in different scenarios. We hope that this simulation study will address your major concern, and convince you of the power of our approach in the particular setting of correcting, in real time, under-reporting of cases during epidemics.

Major comments

1. COMMENT: **As presented the method is somewhat opaque and not sufficiently explored for what I believe is its first application in epidemiology. It is unclear how it performs when incidence is low or high or how it might depend on having a high-quality estimate of the IFR (and on if the IFR is large or small). An extensive simulation study looking at these points with a known ground truth and also better quantifying and calibrating predictive performance against something simple like the ratio of $C(t)/T(t)$ and an existing statistical method (see point 4) is needed. The ground truth could examine several closed-form $T(t)$ models including the constant and more complex ones. These simulations will help clarify and provide insight into what value the proposed methodology adds.**

   This is, indeed and to the best of our knowledge, the first application of symbolic regression to the field of epidemiological modeling and prediction. Therefore, we agree with you that more detailed explanations and validations were necessary. Following your comment, we have now carried out an extensive simulation study, in which we test the ability of the BMS to learn models in: (i) different spreading conditions; (ii) different testing conditions; (iii) different detection conditions. With regards to spreading conditions, we consider four scenarios: a growing epidemic wave, a decreasing wave, a full wave, and two consecutive waves. With regards to testing conditions, we consider two scenarios: one in which testing grows with incidence, and another one in which testing capacity saturates with growing incidence. Finally, with regards to detection, we test scenarios in which the exponent n is constant, and a scenario in which it depends on the epidemic state.

   By combining these, we end up with 16 different simulation scenarios. In all of them, we find that the BMS is able to unambiguously identify the ground-truth testing/detecting conditions,

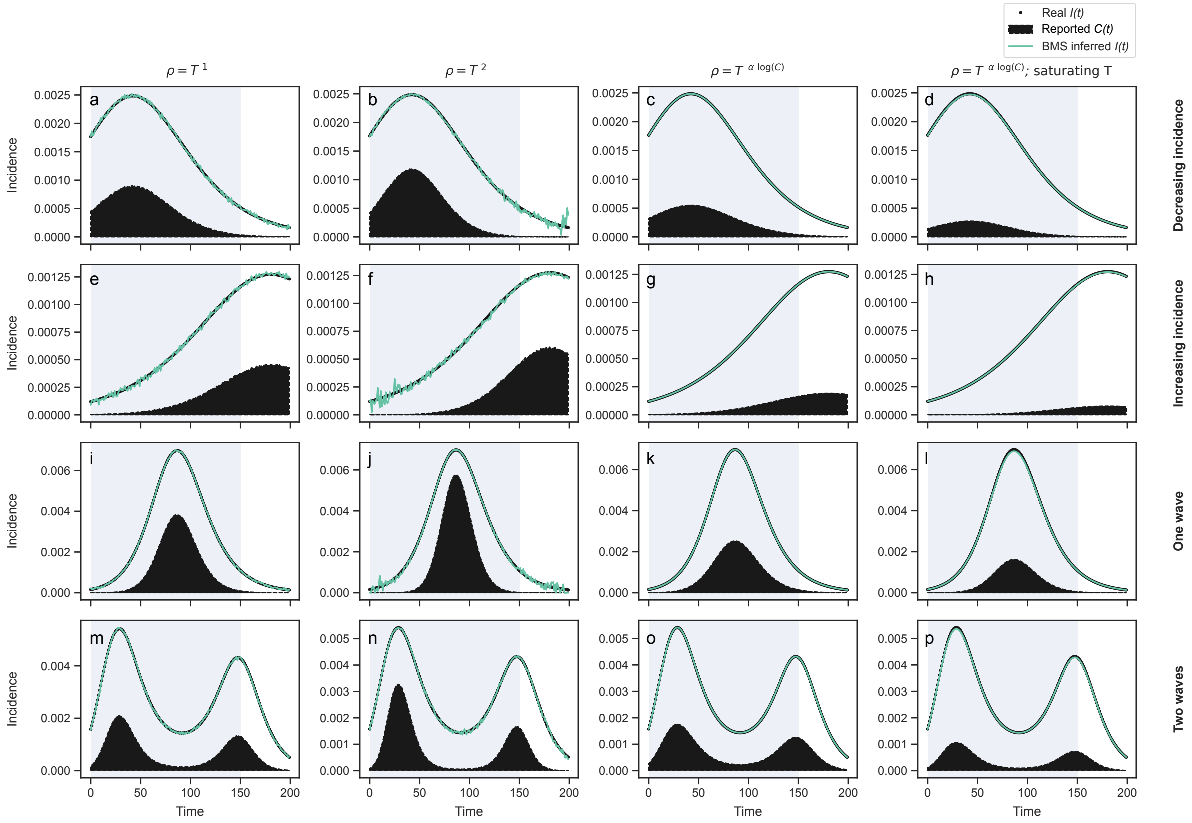

Figure R1: **Estimation and prediction of the incidence $I(t)$ in synthetic data.** We consider four simulated epidemic scenarios: (a-d) decreasing incidence after a peak; (e-h) increasing incidence leading to a peak; (i-l) a complete wave; and (m-p) two consecutive waves. For each of these scenarios, we consider four testing and reporting models (see Methods for details): constant reporting exponent $n = -1$ and testing that grows indefinitely with incidence (left column); constant reporting exponent $n = -2$ and testing that grows indefinitely with incidence (center-left column); variable reporting exponent $n = -\alpha \log C$ and testing that grows indefinitely with incidence (center-right column); variable reporting exponent $n = -\alpha \log C$ and testing that saturates at high incidence values (right column). Black dots represent the ground truth incidence $I(t)$; the black area represents the reported cases $C(t)$; and the green line represents the incidence estimated by the BMS after obtaining a closed-form expression for the reporting exponent $n(C, T)$. We simulate 200 days; we use the first 150 day for training (shaded area) and the last 50 for testing (white area). In all cases, the BMS is able to identify the correct closed-form model for the reporting experiment $n(C, T)$, and thus provides optimal estimates of real incidence.

and thus to make optimal estimations of real incidence (see Fig. R1). These estimates are in real time, in the sense that they can be obtained in terms of daily available data on detected cases $C(t)$ and number of tests administered $T(t)$.

CHANGES: We have now added a whole new section discussing this simulation study.

2. COMMENT: **Using deconvolution with the IFR to obtain a benchmark may be misleading**

on real data (which is also why point 1 is important). The IFR may change with time and phase of the epidemic. Assuming a fixed IFR might then suggest those changes have to be accounted for within the exponent inferred when training the method. This might affect conclusions about whether $n(T, C)$ is 'constant'. This confounder is worth testing and explaining. More generally, the strengths, limits and assumptions of the approach are not well explored and it is hard to gauge its benefits and critique its relative value. More analyses towards this direction would make the manuscript more convincing.

Please note that, in our paper, the deconvolution with the IFR is just a way to estimate real incidence, so that the BMS can elicit models. In general, however, our approach does not depend on the method used to obtain real incidence—all our approach needs is a reasonable estimate of incidence during a certain training period, regardless to the method used to obtain it. In practice, in a new hypothetical pandemic, incidence could be estimated via deconvolution, but also via sentinel testing, serological studies, syndromic surveillance, or any other approach.

That being said, in the present study we were careful to use data prior to the first vaccines were rolled out to the population. The period considered is also relatively short, and close enough to the first wave of the COVID-19 pandemic that reinfection and acquired immunity were still not an issue. Therefore, we see no obvious reason to suspect any systematic changes in the IFR within countries. Additionally, we note that using fatality time series to estimate real incidence, by one method or another, is common practice (not only Goldstein but, as you mention below, in "tens of papers").

Last, but perhaps most importantly, let us assume, for the sake of argument, that IFR changed in some country at some point during the time considered, so our estimates of real incidence are inaccurate, either in the training window, the test window, or both. Then, our predictions would be systematically off the real value, which is not the case[1]. Therefore, again, we see no reason to suspect that there is a problem with our estimation of the real incidence.

CHANGES: We have added a clarification about possible changes in the IFR, and about alternative ways to estimate real incidence for training.

3. COMMENT: **If we require the IFR or death time series to train this algorithm then what is the benefit over simply performing deconvolution and estimating infections that way? This was done with success by several groups starting from the Goldstein *et al* paper already cited and also https://www.nature.com/articles/s41586-020-2405-7 and a number of other papers. This common approach (there are 10s of papers similarly converting deaths to infections) needs to be discussed and the proposed methodology has to add value relative to them. There are also a number of 'nowcasting' approaches that are also worth considering.**

   From your comment, we realize that we were not clear enough in the original submission, and we apologize for that. Death time series or IFR are not required, in principle, to train the BMS. All that is required is *some* estimate of real incidence, which, again, could be obtained via

[1] Also, please keep in mind that the models learned by the BMS only use C and T to make predictions, and in no way require information about fatalities or IFR to make their predictions (see our next response for more on this)

sentinel testing, serological studies or any other approach during initial stages of the pandemic, besides deconvolution of death time series.

More importantly, even if training data are estimated via deconvolution of death time series as in our paper, once the models have been obtained, all that is needed to estimate real incidence are the reported cases C and the fraction of the population tested T on a given day. Since both C and T are typically available on a daily basis, estimations of real incidence can be made in quasi-real time, not *a posteriori* as in Goldstein *et al.* and others. Therefore, after the training period, our approach does allow us to estimate incidence in real time. And, indeed, the test we perform with the empirical data exactly emulates this real-time estimation task—incidence is not used in any way to make preditcions in the test period. Again, we apologize for not making this clear enough in our original submission, which is a fundamental difference with the approaches that you seem to have in mind.

Finally, note that we are also not interested in "nowcasting" the evolution of the epidemic based on, for example, incidence in previous days—we only aim to estimate incidence by correcting reported cases. To the best of our knowledge, the only existing approach to estimate real incidence under these conditions (quasi-real time and based on readily available measures of testing and reported cases alone) is Chiu *et al.*, *PLoS Comput. Biol.*, 2021, over which we show a clear improvement in performance. We feel that this, alone, justifies publication of our work in the same journal were their results were published.

CHANGES: We have thoroughly revised the text to make all of the above considerations clear.

4. COMMENT: **There is little engagement with the literature on the subject of under-reporting and the methods used to understand, correct and quantify its impact. This paper needs to be contextualised against those works to clarify its contribution. Some studies on methods and how performance depends on reporting variability that should be discussed:**

   (a) https://journals.plos.org/plosntds/article?id=10.1371/journal.pntd.0006161

   (b) https://academic.oup.com/aje/article/190/9/1908/6217341?login=false

   (c) https://www.nature.com/articles/s41586-020-03095-6

   (d) https://www.nature.com/articles/s43588-022-00313-1

   (e) https://onlinelibrary.wiley.com/doi/10.1002/sim.6015

   Not only do these contain the common methodology for correcting under-reporting they also discuss how the form of the under-reporting (i.e., how time-varying it is) affects our estimation and prediction performance.

   We thank you for suggesting these references. We agree that, perhaps because our background is mostly in modeling of epidemic spreading and on probabilistic inference, our original submission did not properly contextualize our contribution. In particular, we did not pay enough attention to work dealing with the effects of under-reporting, especially on the estimation of reproduction numbers. We also did not pay enough attention to the emphasis in the literature on the fact (and implications) that the reporting rate is far from constant in time (and space). These are, indeed, important aspects in in the context of our contribution. However, we note that none

of these works deals with correcting for under-reporting in real time based on readily available metrics such as detected cases and number of tests.

CHANGES: We now added a discussion on the consequences of under-reporting for epidemic control and, particularly, for the estimation of reproduction numbers. We have also discussed some of these works in relation to the fact that the reporting rate is not constant during pandemics, which is a central consideration in our work (see our new Fig. 3 and our response to Comment #8 below). Finally, we have discussed how some of these papers report wide disparities in reporting rate, even within single countries, vis a vis our result that a single model for all countries is more parsimonious than country-specific models.

5. COMMENT: **Not enough motivation is provided for why machine learning is needed and what it adds when solving this problem. The stated benefits seem to be accounting for more complex $n(T, C)$ formula and getting closed-form descriptions. Yet the latter, which is meant to help interpretability, are not written explicitly nor are consequences explored. Additionally, the variable of actual interest is $1/b$ and methods exploring complex $1/b$ forms do exist so more work is needed in this direction to distinguish what machine learning is bringing.**

   We find that the actual expressions we obtain for $n(C, T)$ are not easy to interpret. We did attempt to provide interpretation for the expressions, the terms they contain, or even their overall behavior and limits; but, owing to the fact that each country has different parameters, we were not able to draw any general conclusions. This is the reason that, although interpretability is, indeed, one of the strong arguments for symbolic regression, we decided not to emphasize this aspect in our manuscript. Accordingly, nowhere in the manuscript do we claim that the present approach is particularly helpful in this regard.

   That is not to say that our approach does not have advantages or does not provide any insights. First, our approach yields better estimates of real incidence than the model of Chiu *et al.*, *PLoS Comput. Biol.*, 2021. We feel that this, alone, is of enough practical importance. It is also of theoretical importance, as it shows that the assumption $b = 1/\rho = T^n$ with $n = \text{const.}$ is unwarranted and needs careful consideration (ρ here is the reporting rate; see our answer to Comment #8 below).

   Second, the new simulation study shows that our approach is able to identify the true form of the reporting rate $\rho(C, T) = 1/b(C, T)$ (or the exponent $n(C, T)$), when such a closed-form expression exists. This is *asymptotically* guaranteed by the BMS (see Guimera *et al.*, *Sci. Adv.*, 2020, and Fajardo-Fontiveros *et al.*, *Nature Comm.*, 2023, for rigorous justification and discussions), but the new study shows that, in the present context and with typical amounts of available data (weeks to a few months), the true model is indeed identified reliably.

   Last but not least, our approach allows us to test and confirm the hypothesis that a single model for all countries is more parsimonious than different models for each country. No other approach that we are aware of (machine learning or otherwise) would allow us to test this hypothesis. This result is especially remarkable considering that wide disparities in reporting rates have been described in the literature, even within single countries.

   CHANGES: We have added a discussion along these lines in the paper. We have also added figures displaying the reporting rate $\rho = 1/b$, which, we agree, is easier to interpret than b or

the exponent n itself. We have also rewritten the text to refer always to ρ and eliminated b throughout.

6. COMMENT: **There is no mention or attempt to account for delays in reporting cases (which are not negligible). These also add temporal components to the reporting rate (see again some of a-e above) and the impact of this related noise source needs to be explored for any analysis on real data. Delays in reporting may be adding some of the complexity to $n(T, C)$ and are extremely important for correcting cases to infections (also see nowcasting). A related paper is https://onlinelibrary.wiley.com/doi/abs/10.2307/3315826.n1**

   We are not entirely clear whether you are referring to delays in reporting C and T, or delays between infection and detection (and, thus, reporting) of cases. Neither of them are major problems for our approach, although we agree with you that more discussion was needed in this regard.

   Delays in reporting C and/or T are not a major issue nowadays. Indeed, during the COVID-19 pandemic (except, perhaps, the first few weeks of the first wave), C and T were reported daily and with little error in most developed countries; numbers changed little after $C(t)$ and $T(t)$ where first published on day t. In any case, possible delays in reporting C and/or T could be accounted for as discussed in Lawless, *Canad. J. Stat.*, 1994. We thank you for suggesting this reference.

   The delay between infection and detection also has relatively mild effects on our approach. Although it is obvious that cases are detected a few days after infection, if the time between infection and detection is sufficiently narrowly distributed (which it is for many infectious diseases, and COVID-19 in particular), then both curves are just shifted in time. The need for a time "correction" would be evident if the curves $C(t)$ and $I(t)$ used for training were clearly and systematically shifted. In any case, we do not implement any such shifting, because our $C(t)$ and our $I(t)$ estimated by deconvolution are not significantly and consistently shifted.

   CHANGES: Following your comment, we have added to the manuscript a discussion on reporting delays and their possible consequences on our method. We have considered both possible sources of delay (in reporting C and T, as well as between infection and detection), and added references as suggested.

7. COMMENT: **The value of using the minimum description length (MDL) is unclear. With the Laplace approximation it becomes a BIC with a prior penalty. How would performance fair if we simply use the BIC? Other papers apply MDL to epidemics via Fisher approximations (https://academic.oup.com/sysbio/article/69/6/1163/5825296?searchresult=1) or proper scoring rules (https://www.ncbi.nlm.nih.gov/pmc/articles/PMC6386417/), which amount to the equivalent. These also need to be discussed for context else the results about parsimony do not seem that new and are hard for unaware readers to parse.**

   The value of using Bayesian model selection (and description length) has been discussed and justified in Guimera *et al.*, *Sci. Adv.*, 2020, and the optimality of the approach has been studied in depth in Fajardo-Fontiveros *et al.*, *Nat. Comm.*, 2023. Therefore, we feel that it is not necessary

(or, indeed, possible) to cover this again in the present manuscript. In any case, please note that our approach results from very basic (and explicit) probabilistic considerations, and therefore it is not heuristic or arbitrary. In particular, we do not choose to use description length; description length (with a BIC term and a "penalty") is what we obtain from our calculations.

To answer your specific question: Simply using the BIC for model selection in the context of symbolic regression is inappropriate because it leads to what we called *structural overfitting* in Guimera *et al.*, *Sci. Adv.*, 2020. Structural overfitting arises from the fact that there are exponentially more complex closed-form models than simple closed-form models, so one can always find very complex models that make the BIC arbitrarily small. In this sense, structural overfitting is akin to "traditional" overfitting, but arises from the addition of terms to expressions instead of the addition of parameters. Like traditional overfitting, structural overfitting results in very poor extrapolation to unseen data.

Finally, we note that probabilistic and minimum description length approaches are standard, much in the same way that many people may use maximum likelihood approximations or any other statistical methodology. Keeping this in mind, we see no clear relationship between our use of probabilistic approaches for model selection and symbolic regression, and previous uses of MDL methods in epidemiology (much in the same way that two papers using maximum likelihood in epidemiology are not necessarily related).

CHANGES: We have clarified this in the text succinctly.

8. COMMENT: **It would be good to convert the exponents in Fig 1 back to the actual rate of reporting ($1/b$, which is what is really of interest) and in Fig 2 to include $C(t)$ for reference.**

   We completely agree and thank you for this suggestion.

   CHANGES: We have edited our manuscript throughout to write everything in terms of the reporting rate $\rho = 1/b$, added a figure for ρ (new Fig. 3) and added $C(t)$ to incidence plots, both in the simulation study and in the empirical study. Although we prefer to maintain the sign of the exponent as $1/\rho = T^n$ for consistency with Chiu *et al.*, we now make all discussions in terms of the rate of reporting.

9. COMMENT: **Last, is there any data for the case studies chosen to show what the best estimate of the infections in those regions are (versus the deconvolution that depends on IFR)? There should be for France at least. If it can be shown relative to those best estimates that this approach outperforms others as noted also in earlier points, this would strengthen the paper and help with interpretation of the Figs as e.g., it is unclear if the estimates for UK, Hungary, France and Catalonia are that good in absolute terms.**

   To the best of our knowledge, there is no ground truth about the number of cases in the period and countries considered—short of testing the entire population frequently, all we can possibly have are estimates of real incidence, which are always subject to modeling and estimation assumptions. Estimates of real incidence in France that are likely very accurate (because of the combination of approaches that were used to obtain them) are available from Pullano *et*

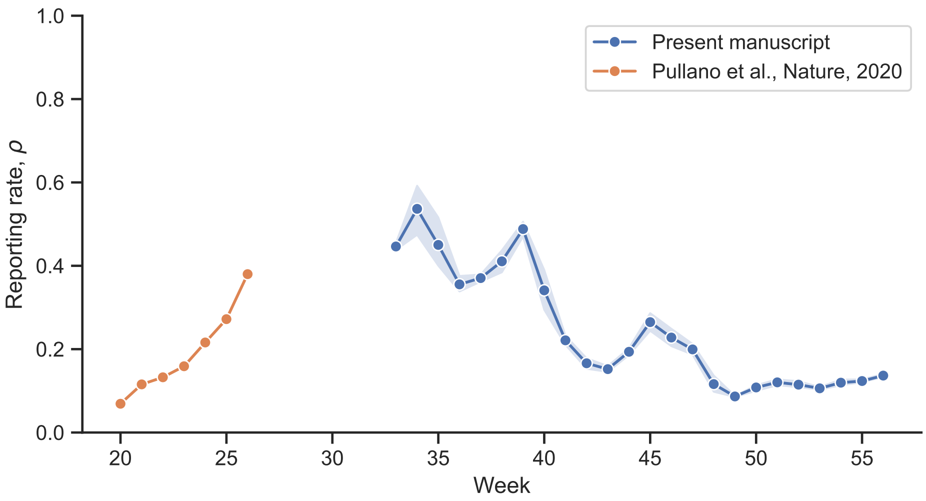

Figure R2: **Comparison of estimates of the reporting rate.** Reporting rate ρ in France from Pullano *et al.*, *Nature*, 2020 (Fig. 3a in there) and ours. Although the time windows do not overlap, the estimates are compatible. Weeks are counted from Jannuary, 2020, for consistency with their paper.

al., *Nature*, 2020; unfortunately, the period studied there does not overlap with the period we have considered here. However, their estimates of the reporting rate and ours are compatible (Fig. R2).

That being said, we note again that:

- As suggested by Goldstein *et al.* and many others, deconvolution from deaths is a very natural way to estimate real incidence, and hardly contentious when, as discussed above, there is no reason to suspect changes in IFR.
- Ultimately, our method for correcting under-reporting does not rely on any particular approach to estimate real incidence—given *any* such estimate (deconvolution or otherwise), as well as data about testing and reported cases, we can model the rate of reporting.
- The models that we obtain for the rate of reporting allow us to estimate real incidence in the test set (that is, outside of the training period), from testing and reported cases alone; that is, without any reference to deconvolutions. If either incidence estimation or the models were inadequate, the out-of-sample test would give poor results. However, we obtain results that are consistently superior to those of Chiu *et al.*

Reply to Reviewer #2

COMMENT: **This is a review of "Machine learning mathematial models for incidence estimation during pandemics" from Fajardo-Fontiveros and colleagues, submitted to *PLOS Computational Biology*. The big idea in this paper is to use Bayesian symbolic regression (which the authors call a Bayesian Machine Scientist, BMS) to learn not only parameters (as we might in a typical Bayesian modeling problem) but a function as well. While BMS is described elsewhere, here the authors ask: can we use it to learn about the underreporting of COVID-19 infections? They express underreporting as a factor b, defined as the fraction of total infections (I) which are reported as cases (C), and the BMS specifically provides a posterior over functions describing how b scales with the fraction of the population being tested.**

The technical details of how the BMS works are provided (and developed in detail in Ref 15), but for the purposes of this paper, the authors really do two things: First, they fit both nation-specific and global models and ask which provides a better fit after accounting for model size (via rigorous model selection). [Answer: a global model is more parsimonious.] Second, they show that (1) the relationship between the underreporting factor b and the amount of testing T varies over time, and (2) a time-varying relationship between b and T leads to good predictions of incidence. There are a few issues with the paper that hold it back, which I think could be addressed, but might require more changes than the authors had in mind. They follow, below.

We thank you for reading our manuscript and for your constructive criticism. Your summary of our results is accurate and highlights what we feel are main contributions to the state of the art. In this regard, however, we would like to add what perhaps is the most important practical contribution of our work—after the initial training, our approach allows us to correct under-reporting in real time. In what follows, we clarify some misunderstandings that seem to have arisen due to lack of precision on the original manuscript, and that are at the heart of your concerns.

1. COMMENT: **Fundamentally, this paper is framed about solving one problem, but then goes on to solve a different problem. What I mean is: the authors make a reasonable case that this method provides a means to retroactively adjust case counts to estimate true incidence. However, the framing of the paper is about real-time measurement of incidence for the purposes of control. I don't see results that evaluate a real-time prediction. Instead, they use 4 months of training data (Aug, Sept, Oct, Nov 2020) to predict two months of test data (Dec 20, Jan 21). The models also require mortality, case, and testing (T) data on which to train, but I don't think these are actually available in real time.**

   Please note that our method *does* allow correction of under-reporting *in real time*; that is, it allows us to estimate real incidence $I(t)$ at time t from reported cases $C(t)$ and testing $T(t)$ at time t. We apologize for not making this clear enough in our original submission, and try to explain it better here and in our revised manuscript.

   The BMS, as any supervised statistical learning method, requires training data. In the present study, these training data consist of three time series: the target $I(t)$, and the features $C(t)$ and $T(t)$. During the COVID-19 pandemic, $C(t)$ and $T(t)$ were available for most countries on a daily basis. By contrast, $I(t)$ is typically not available at time t—of course, this is precisely what

we aim to address with our method. Therefore, for training, we resort to the deconvolution of downstream deaths to retrospectively reconstruct $I(t)$. By using this approach, you are correct, training cannot be done in real time.

However, the key observations with regards to prediction are the following:

- Once the BMS has identified models $I(t) = T(t)^{n(T(t),C(t))} \times C(t)$ (that is, after training), all that is needed to estimate real incidence $I(t')$ at day t' are the values of $C(t')$ and $T(t')$ on that same day. These, again, have been available daily for most countries during the COVID-19 pandemic (except perhaps the first few weeks of the first wave), which means that $I(t')$ could be estimated daily, in real time.
- In this sense, our test on data from December 2020 and January 2021 is exactly as if predictions were made in real time. Please note that, in the context of the test, deconvolution of deaths is only needed to quantify the accuracy of our incidence predictions, but not to make the predictions themselves.
- Strictly speaking, and somewhat tangencially to your comment, deconvolution is not necessary for our approach—all that is needed is some training series $I(t)$. Whether this series is obtained by deconvolution of by some other method (for example, sentinel testing or serological studies) is irrelevant.

CHANGES: We have clarified this in the manuscript.

2. COMMENT: **Would this work on other infectious diseases, or ILI? The Goldstein deconvolution approach was developed for flu if I recall correctly... In any case, if the authors hope to argue that the BMS approach here is going to be useful for future pandemic control, then it would be great to be able to make that case on less time-restricted and pathogen/variant-restricted data.**

   Goldstein's deconvolution approach was developed for reconstructing infections during the 1918 influenza epidemic in the US, and there is no reason why it should not work for other infectious diseases. However, we note that:

   - For diseases such as seasonal flu, with much lower IFR than the 1918 influenza or COVID-19 and resulting in at least one or two orders of magnitude fewer fatalities, incidence estimation by deconvolution is more prone to low-number errors and fluctuations.
   - For diseases such as Ebola, which cause much more severe symptoms and with IFR much higher than the 1918 influenza or COVID-19, most cases are detected anyway (at least after an initial phase in which detection is not exhaustive), so $I(t) \approx C(t)$ and there is typically no need to estimate incidence beyond reported cases (see Dalziel *et al.*, *PLoS Neglected Tropical Diseases*, 2018).

   Perhaps because of this, we are not aware of any detailed databases with daily reported cases, testing, and mortality data (or any other data that could be used to estimate daily incidence) that we can use.

   That being said, we note that we are already studying COVID-19 in several different countries, with different socioeconomic makeups, and with different control and mitigation strategies.

Moreover, we see no theoretical argument as to why our approach may not work in other infectious diseases or situations. After all, our approach is based on the theoretical guarantees of the BMS (see Fajardo-Fontiveros *et al.*, *Nat. Comm.*, 2023).

To confirm this, and lacking comparable real data on other diseases, we have now added to the manuscript an extensive simulation study with synthetic data and known ground truth. In this study, we test the ability of the BMS to learn models in: (i) different spreading conditions; (ii) different testing conditions; and (iii) different detection conditions. We find that the BMS always recovers the ground truth, and gives optimal estimations of real incidence (see Fig. R1).

CHANGES: We have added to the manuscript a brief mention of the potential applicability of our method to other diseases. We have also added a simulation study showing that, when the relationship between I, T and C is known, the BMS has no difficulty in identifying it and providing excellent estimates of real incidence.

COMMENT: **What are the limitations of this approach, data, conclusions, etc? Relatedly, is Goldstein really the right ground truth? Are there alternatives? How could this model work on *actual* real time data, knowing that many of the datastreams during the acute phase of the pandemic were delayed by varying amounts (or published by reporting data -vs- swab date, etc)? Relatedly, are there other extensions that could be discussed?**

With regard to these questions, we note the following

- Strictly speaking, there is no ground truth about the number of cases in the period and countries considered—short of testing the entire population frequently, all we can possibly have are *estimates* of real incidence, which are always subject to modeling and estimation assumptions.
- Considering this, and as mentioned in our previous replies, Goldstein seems to provide a reasonable "ground truth" in practice. In any case, our approach is not dependent on fatality deconvolution—it could work on any time series of estimated real incidence (obtained, for example, from sentinel testing or serological studies).
- As also mentioned above, and to circumvent the lack of real ground truth in an empirical setting, we have conducted our simulation study with synthetic ground truth, with excellent results in all cases.
- Delays in reporting C and/or T are not a major issue nowadays. Indeed, during the COVID-19 pandemic (except, perhaps, the first few weeks of the first wave), C and T were reported daily and with little error in most developed countries; numbers changed little after $C(t)$ and $T(t)$ where first published on day t. In any case, possible delays in reporting C and/or T could be accounted for as discussed in Lawless, *Canad. J. Stat.*, 1994, as suggested by Reviewer #1, without altering any substantive aspects of our approach.
- Given the previous observation, we argue that there is very little difference, if any, between our validation study on test data, and a test on "actual real-time data."

CHANGES: We have added discussions about each of these issues in the manuscript.

3. COMMENT: **Are the functional forms in the country specific OR global models interesting or useful? Is the composition of each country's posterior the same, in terms of the functional forms we see? In other words, can we learn anything about the actual samples from the BMS posterior that teach us something about surveillance, infectious diseases, testing, etc? Or is this [simply] a powerful black-box method, and we shouldn't try to look under the hood to learn more? As written on Line 122, the model selection findings, showing that a common mechanism for underreporting is the more parsimonious model, raise "questions about the nature of such mechanisms," but the authors have missed an opportunity to help answer or discuss said mechanisms.**

   As shown by the simulation study, with an amount of data similar to the empirical datasets, the BMS has no difficulty in identifying the true closed-form models, when these exist. However, we find that the actual expressions we obtain for $n(C, T)$ from the empirical data are not easy to interpret. We did attempt to provide interpretation for the expressions, the terms they contain, or even their overall behavior and limits; but, owing to the fact that each country has different parameters, we were not able to draw any general conclusions. This is the reason that, although interpretability is one of the strong arguments for symbolic regression, we decided not to emphasize this aspect in our manuscript.

   That is not to say that our approach does not have advantages. First, our approach yields better estimates of real incidence than the model of Chiu *et al.*, *PLoS Comput. Biol.*, 2021. We feel that this, alone, is of enough practical importance. It is also of theoretical importance, as it shows that the assumption $b = 1/\rho = T^n$ with $n = \text{const.}$ is unwarranted and needs further consideration. Additionally, our approach allows us to test and confirm the hypothesis that a single model for all countries is more parsimonious than different models for each country. No other approach that we are aware of (machine learning or otherwise) would allow us to test this hypothesis. And we find the conclusion especially remarkable, considering that the literature has emphasized the variability of under-reporting, even within single countries (see, for example, Dalziel *et al. PLoS Negl. Trop. Dis.*, 2018; Pitzer *et al.*, *Am. J. Epidem.*, 2021; Pullano *et al.*, *Nature*, 2021).

4. COMMENT: **In conclusion, I enjoyed reading this paper, and it's a nice idea with some new technology for the readership of PLOS Comp Bio. But, the paper feels underdeveloped. We learn that a previously developed hammer (BMS) can hit this particular nail (2020-2021 COVID-19 undereporting), but don't learn much about the hammer, this class of nail, other similar nails, and so on. The paper has a lot of potential, but without unpacking more about what the BMS *means*, and what it *tells us*, it could easily be any other black-box ML approach—with the exception of the model selection result. Unfortunately, the model selection result (one model, not lots of country-specific models) is not enough, in my opinion, to hang the paper on.**

   We thank you again for reading the manuscript, for your praise about the ideas in our work, and for your comments and constructive criticism. However, we feel that it is somewhat unfair to claim that our paper hangs on a single result. Rather, we identify at least four significant contributions:

- We provide real-time incidence estimates that are more accurate than Chiu *et al.*, which was published precisely in *PLoS Computational Biology*. We are not aware of other approaches that estimate real incidence in real time from reported cases and number of tests alone.
- We prove that one key assumption in Chiu *et al.* ($n = \mathrm{const.}$) is not warranted.
- We show that, despite the breakdown of this assumption, the idea that I can be estimated from C and T using $I = T^{n(C,T)} \times C(t)$ is valid and provides good incidence estimates.
- We prove that a single model for all countries is more parsimonious that a different model for each country. No other approach that we can think of (machine learning or otherwise) could have lead to such a conclusion. Again, we find the conclusion remarkable, considering that the literature has emphasized the variability of under-reporting, even within single countries.

We hope that, after our answers, the new simulation study, and the changes to the manuscript, you can now conclude that the paper is suitable for the readership of *PLoS Computational Biology*.
