# Supplementary material for: Machine learning mathematical models for incidence estimation during pandemics
Source: PLoS Comput Biol. 2024 Dec 23;20(12):e1012687. doi: 10.1371/journal.pcbi.1012687 (PMC11706490; doi:10.1371/journal.pcbi.1012687)
Supplement: Attachment — Submitted filename: Incidence_estimation_REBUTTAL.pdf [file pcbi.1012687.s002.pdf]

Reply to Reviewer #1

COMMENT: **Many thanks to the authors for a detailed and meticulous reply. I think the manuscript is much improved. However, I am still left unconvinced about the added value of the approach. Some reasonable additions to the new simulations and some qualifications could resolve this.**

We are happy that the reviewer finds the manuscript much improved. We think that our additions to the simulations and our editing of the manuscript ought to dispel any remaining questions.

COMMENT: **The authors call these real-time estimates. But if you require 150 days for training, it is only real time after quite a substantial 'burn-in'. After 150 days it may be too late for these analyses. Can the authors qualify what minimum level of training is needed or explain this issue?**

We note that: (i) any supervised method will require a training period; (ii) for the COVID pandemic, the 150 days we consider represent only one wave, and in some cases not even that (Canada, Croatia, Hungary, Italy). That being said, we have now tested, with the synthetic data, how many days of training are necessary to recover the exact models that generated the data. We find that, in all scenarios considered previously, 40 days of data are enough to recover the ground truth.

CHANGES: We have updated Fig. 1 in the manuscript to reflect the new experimental setup, in which only 40 days are used for training.

COMMENT: **Fig R1 – I like this figure but:**

1. **Please include C(t)/I(t) where I(t) is either from the simulation or estimated from deaths. In short, while the shown predictions do look good, without a reference for comparison it is hard to understand if they are improved in some sense.**
2. **Following on from that, a comparison against another method e.g. maybe just a simple statistical time series model. It is not that I disbelieve the approach, just that I want to understand what it improves upon to justify the increased complexity.**

Following the reviewer's suggestion, we already included Fig. 3 in the last revision of our manuscript, which shows the reporting rate $\rho = C(t)/I(t)$ for the real data estimated from deaths. In that figure, and throughout the manuscript, we also compare with the model by Chiu *et al.*. Additionally, especially from Fig. 3 but also from other figures in the manuscript, it should be clear that other simple models such as $\rho = \mathrm{const.}$ are also poor descriptions of the data. We acknowledge that these representations have made our case stronger, and thank the reviewer for the suggestions.

However, we note that the purpose of Fig. 1 is different—to show that, when a closed-form expression exists for the reporting exponent, the BMS is able to find it. The fact that the corrections to reported cases are optimal in this case, and thus that incidence can be estimated very precisely, is just a consequence of the fact that we recover the correct model exactly. From this perspective (and also considering the flow of ideas within the manuscript), we find that it would be confusing to introduce other models for comparison in Fig. 1.

For all the reasons above, we would much rather not complicate the figure further, and we prefer to leave it as is (except for changing the training period from 150 to 40 days, as explained in our previous reply).

COMMENT: **Is the Chiu et al method simply the constant exponent? It isn't made that clear what they do and if the authors base their results on being more accurate than this, then it should be made very clear what Chiu et al propose. In the paper Chiu et al is only mentioned once. Are Chiu et al using symbolic regression or another method? Please clarify.**

Chiu *et al.* do not use symbolic regression or any other statistical learning method—they just propose a rationale for (and then assume and test) the model $\rho = T^{-n}$, with being n a constant, country-specific exponent.

CHANGES: We have expanded our explanation on the rationale and model of Chiu *et al.*.

COMMENT: **As the authors note any reasonable estimate of incidence is needed for training and hence this does not depend on good IFR estimates and deconvolution. I have no issue with that, my concern was that if I had a good IFR and inferred infections from it (which is now a common approach) then what benefit do I gain from applying the authors' method? This is the question that still needs addressing.**

We already addressed this concern in our previous reply. After the training period, given the number of tests and of reported cases on a day, we can immediately estimate the real incidence on that day. By contrast, to estimate real incidence by deconvolving deaths with the IFR, one needs to wait several weeks.

COMMENT: **The authors state that delays in reporting are not a concern. The field of nowcasting exists precisely because it is a notable problem (and even more substantial one for deaths), even in high-income countries. While reported cases appear daily, as the authors note, the cases that are reported are not all the cases that should be counted on that day. This is due to such delays, which for COVID were above 5 days. Please ensure the text acknowledges this issue.**

We disagree that the field of nowcasting and approaches such as ours exist "precisely" because of delays—they exist mainly because, in contexts such as the COVID-19 pandemic, and regardless of time, most cases go unreported.

That being said, we already addressed the issue of delays in our previous reply. With regards to the delay between infection and detection, we noted that, if the time between infection and detection is sufficiently narrowly distributed, then the infection and detection curves are just shifted in time (if the distribution were not so narrow, then there would be a smoothing of the detection curve, in addition to the shift). In the case of COVID-19, infection-to-detection time was narrowly distributed around a mean of $\sim$5 days.

In our previous reply we also noted that, in any case, the need for a time correction would be evident if the curves $C(t)$ and $I(t)$ used for training were clearly and systematically shifted, which is not the case.

CHANGES: We have added a more nuanced discussion of this issue to the manuscript.

COMMENT: **Fig R2 – I am unsure what the estimates being compatible means.**

By this we just mean that, although time windows do not overlap and therefore our estimates and those previously reported are not directly comparable, they are certainly not contradictory. First, the ranges of estimated reporting rates are very similar. Second, the overall tendencies (growth of the reporting rate up to week ∼30, followed by an overall decrease afterwards) seem plausible; in particular, the last estimate in the literature (week 26) and our first estimate (week 33) are very close. We reproduce the figure here again for completeness.

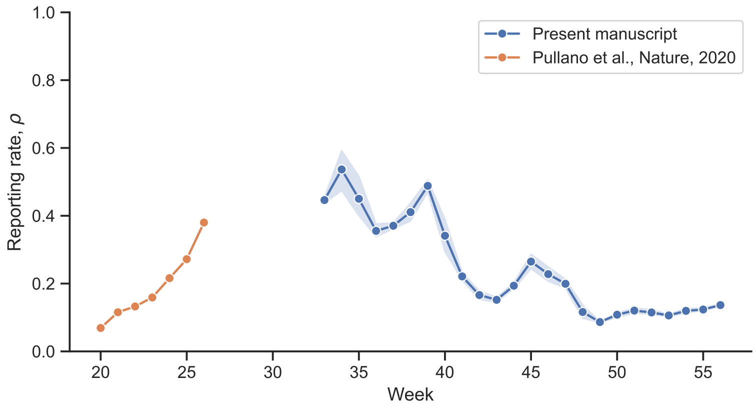

COMMENT: **Last, what is the interpretation behind why the authors believe a single model across countries is beneficial over the multiple models? This could be useful for readers.**

Our interpretation of this finding is that, despite notable differences between countries, there are some common mechanisms leading to detection and reporting of cases. Then, the pooling of the data of all countries into a single dataset for training improves the learning process, in the sense that each country's data helps in the modeling of other countries.

CHANGES: We have expanded our discussion of this issue in the manuscript, speculating about possible common mechanisms.

Reply to Reviewer #3

COMMENT: **Authors are advised to include the recommendation of future work.**

We are happy that all previous concerns have been dispelled.

CHANGES: We have pointed out a few possible venues for future work in the discussion.

Reply to Reviewer #4

COMMENT: **The points raised by the reviewers in the first round of the review have been satisfactorily addressed in the current version of the manuscript. The manuscript now provides stronger evidence for the robustness, accuracy, and it engages more deeply with relevant literature, improving its clarity and theoretical grounding.**

The introduction offers a comprehensive background, particularly focusing on the challenges of under-reporting during pandemics, and how this impacts real-time incidence estimation. It outlines the motivation for developing a machine learning model to correct under-reporting based on reported cases and testing data.

The reasons for performing the study are clearly defined and the study objectives are clearly articulated. The methods used in the study are generally appropriate for the objectives of estimating real-time incidence during pandemics. The use of Bayesian symbolic regression and machine learning techniques to derive closed-form mathematical models is well-suited to address the challenge of under-reporting, and the study's focus on testing the model across various pandemic scenarios (using synthetic and real-world data) is robust. Additionally, the choice to explore whether a single unified model can apply across multiple countries seems innovative and relevant to the study's goals of generalizability.

We thank the reviewer for their careful reading of our manuscript, for their constructive comments, and for their positive assessment and recommendation to publish our work.

COMMENT: **The manuscript provides much of the necessary information for replication, including details of the datasets used, the Bayesian Machine Scientist (BMS) method, and the epidemic models tested in the simulation study. However, some of the technical details regarding model selection, training procedures, and hyperparameter choices for the machine learning algorithms could be expanded. For instance, a more detailed description of the parameters used in the Bayesian symbolic regression or the specific implementation details of the epidemic models (e.g., the equations governing the synthetic data generation) would ensure that a researcher can fully replicate the experiments. Additionally, including code or detailed pseudocode could further improve reproducibility.**

Details on the use of the BMS are provided in the Methods subsection "Bayesian machine scientist". Details on the implementation of the epidemic models used to generate synthetic data are provided in the Methods subsection entitled "Validating the Bayesian machine scientist (BMS) through compartmental epidemic models" (see also Tables 2 and 3). Code for the BMS is open a freely available from `https://bitbucket.org/rguimera/machine-scientist/`.

CHANGES: We have revised the Methods section, and expanded it where necessary, to guarantee that all aspects of the analysis are described in sufficient detail so as to be reproducible. We have also added a link to the BMS code.

COMMENT: **The manuscript includes appropriate references when discussing established methods for under-reporting correction and machine learning. The authors refer to key works, such as Chiu et al. and the Goldstein deconvolution approach, and they also cite relevant literature on Bayesian symbolic regression. The manuscript includes mean absolute error (MAE) metrics to quantify prediction errors, which are easy to interpret.**

We are happy that the reviewer finds our framing and evaluation metrics appropriate.

COMMENT: **The manuscript touches on some limitations, such as the reliance on reported case and testing data and the assumption that the infection fatality rate (IFR) remains constant over time. However, the discussion of limitations could be expanded. Major limitations that should be**

addressed include: (i) potential overfitting to specific pandemic scenarios; (ii) The implications of incomplete or delayed testing data.

Because of the rigorous probabilistic treatment implicit in the BMS, it treats uncertainty optimally and does not overfit the data. However, it is true that it will learn models suitable do describe specific pandemic scenarios, and probably not applicable to others. That being said, the fact that we identify a single model for all countries (albeit with different parameter values) and all times suggests that the models identified are indeed quite expressive.

With regards to incomplete or delayed testing data, as we discussed in our first rebuttal (and here, above), if the time between infection and detection is sufficiently narrowly distributed, then the infection and detection curves are just shifted in time (if the distribution were not so narrow, then there would be a smoothing of the detection curve, in addition to the shift). In the case of COVID-19, infection-to-detection time was narrowly distributed around a mean of $\sim$5 days. In our previous reply we also noted that, in any case, the need for a time correction would be evident if the curves $C(t)$ and $I(t)$ used for training were clearly and systematically shifted, which is not the case.

CHANGES: We have elaborated and provided more detail on these issues in the manuscript.

COMMENT: **Based on the review provided, the following points should be explored in this new round of revisions:**

1. **Clarification of Technical Details for Replication;**
2. **Expansion of Limitations;**
3. **Additional Reproducibility Considerations;**

After these points are addressed, the reviewer recommends that the manuscript be accepted for publication.

We thank again the reviewer for their constructive suggestions and for recommending that the manuscript be accepted.

CHANGES: As detailed above, we have revised the Methods section, and expanded it where necessary, to ensure reproducibility; we have added a link to the BMS code; and we have discussed in more depth the issues of time delays and of overfitting to specific pandemic scenarios.

Reply to Reviewer #5

COMMENT: **The revised manuscript presents an approach utilizing Bayesian symbolic regression to estimate the real-time incidence of infectious diseases, focusing on COVID-19. Below is a detailed review of the key strengths and areas for improvement in this second-round submission.**

Strengths:

1. **The manuscript addresses a critical issue in epidemic management: underreporting due to limited testing, especially during pandemics. The potential application to other infectious diseases strengthens the paper's relevance beyond the immediate context of COVID-19.**

2. **A comprehensive simulation study evaluating the approach across different epidemic scenarios (e.g., growing, decreasing, and two-wave epidemics) provides solid evidence for the method's validity. The BMS's ability to recover the true reporting model across varying conditions is an important contribution.**

We thank the reviewer for their careful reading of our manuscript and for their positive assessment of our work.

COMMENT: **Drawbacks:**

1. **While the novel application of symbolic regression is a strength, the manuscript would benefit from further clarification on what unique contributions this method brings beyond existing nowcasting and underreporting correction approaches.**
2. **One limitation noted in the manuscript is the difficulty interpreting the closed-form expressions derived by BMS. The paper mentions that efforts to interpret these expressions were not fruitful due to parameter variability across countries. While interpretability is not always required for machine learning models, symbolic regression is often praised for this ability, which is not emphasized here.**
3. **A previous review raised concerns about reporting delays, which are not sufficiently addressed in the current version. While the authors argue that these delays were not major during the COVID-19 pandemic, they should still be explored.**

We thank the reviewer for their constructive comments. Below, we provide answers to each of these issues and/or a summary of changes introduced in the manuscript.

COMMENT: **Expand the discussion on BMS's relative advantages over traditional deconvolution methods. Highlight BMS's unique interpretative power or computation speed.**

As noted earlier, deconvolution methods do not allow for real-time estimation of real incidence. With regard to real-time correction methods, Chiu *et al.* is, to the best of our knowledge, the state of the art. With respect to Chiu *et al.*, our method makes more accurate predictions, and shows the limitations in the assumptions made there. Besides, our approach allows us to test the hypothesis that a single model can describe all countries simultaneously, which we confirm.

CHANGES: We have emphasized these considerations in the manuscript.

COMMENT: **Consider providing simplified examples of interpretable symbolic regression outputs, even if these are less complex models. This would help demonstrate the potential for insight extraction beyond predictive power. If interpretability is not a central goal, this should be clearly stated.**

CHANGES: We have clearly stated in the discussion of the manuscript that, despite interpretability being a potential benefit of symbolic regression, here it is not a central goal, given the complexity of the expressions identified by the BMS. We have edited this section for clarity.

COMMENT: **Provide a more detailed discussion of how reporting delays (both in test results and case reports) could influence the results and whether corrective strategies, such as time-shifting the data.**

As we discussed in our first rebuttal (and here, above), if the time between infection and detection is sufficiently narrowly distributed, then the infection and detection curves are just shifted in time (if the distribution were not so narrow, then there would be a smoothing of the detection curve, in addition to the shift). In the case of COVID-19, infection-to-detection time was narrowly distributed around a mean of $\sim$5 days. In our previous reply we also noted that, in any case, the need for a time correction would be evident if the curves $C(t)$ and $I(t)$ used for training were clearly and systematically shifted, which is not the case.

CHANGES: We have elaborated and provided more detail on these issues in the manuscript.
